# An exhaustive scrutiny to amplify the heating prospects by devising a core@shell nanostructure for constructive magnetic hyperthermia applications

**DOI:** 10.1038/s41598-023-39766-3

**Published:** 2023-08-22

**Authors:** S. P. Tsopoe, C. Borgohain, Manoranjan Kar, Shantanu Kumar Panda, J. P. Borah

**Affiliations:** 1 Department of Physics, National Institute of Technology Nagaland, Dimapur, Nagaland 797103 India; 2 Central Instrumentation Facility (CIF), Indian Institute of Technology Guwahati, Guwahati, 781039 India; 3 Department of Physics, Indian Institute of Technology Patna, Patna, Bihar 801106 India

**Keywords:** Cancer, Nanoscience and technology, Physics

## Abstract

An interfacial integration at the nanoscale domain through a core@shell (CS) nanostructure has constructively unbarred a wide dimension to researchers on biomedical applications, especially for magnetic fluid hyperthermia. Lately, the interconnection of the exchange bias effect (EBE) through the interface coupling to the magnetic heating efficiency has uttered its utmost prominence for researchers. Here, we delineate the ascendency of the heating ability through a coalescing assembly of mixed ferrite Co_0.5_Zn_0.5_ Fe_2_O_4_ (CZ) and soft magnetic material Fe_3_O_4_ (F), by devising a network of CoZnFe_2_O_4_@Fe_3_O_4_ (CZF) CS nanostructure. A hefty interface activity with validation of the EBE phenomenon is divulged through magnetic scrutiny for the CS sample. The magnetic nanoparticles heating response to applied magnetic field and frequency is discerned at three distinct fields, where the outcome prevailed to inflated specific loss power for CS CZF in distinction to bare F and CZ samples for all the assessments. Remarkably; a lofty intrinsic loss parameter is also perceived for the CS sample recorded to about 5.36 nHm^2^ g^−1^; which is another eccentric outcome that significantly labels the CS CZF sample as a potentially high heating competence agent. This comprehension accords to a finer perspective to meliorate the theranostic environment for hyperthermia applications.

## Introduction

Single-domain magnetic nanoparticles (MNPs) have become one of the potentially explored research which has gained gigantic attention in the field of biomedical applications^1,2^. Hyperthermia, a cancer remedy through the heat generation on suspension of the MNPs in the tumor cells via AC magnetic field is one of the emerging fields of biomedicine applications. Unlike radiotherapy or chemotherapy, localized and selective targeting heating of tumor cells is designed to even at low dosages of MNPs for hyperthermia which is carried out at shorter time regimes as well. Higher dosages of MNPs will regard to toxicity which results in potential side effects for directed cancer therapy. Hyperthermia efficiency is entirely associated with specific loss power (SLP), which regards the quantity of heat released by MNPs per unit mass. Therefore, the therapeutic need is to secure a maximum SAR value with minimal concentration that will outstretch to threshold temperature (42–46 °C)^3–6^. Indeed, this therapy using MNPs has been effectively demonstrated in several cancer treatments such as prostate cancer, brain tumor, invasive breast carcinoma, etc^7,8^. The past investigation prevailed that soft MNPs are preferable within the clinical limit of applied field and frequency for effective hyperthermia^9^. Iron-oxides MNPs are the most investigated materials for this cancer therapy, especially Fe_3_O_4_ and γ-Fe_2_O_3_ are proven to be non-toxic even at 1 mg/mL concentration which is competently tolerated by human tissues^10–12^. However, despite the non-toxic property etiquette of Fe_3_O_4_ and γ-Fe_2_O_3_, their low SLP values and less chemical stability make them unpleasing for effective magnetic hyperthermia applications. Therefore, one must emphasize to improving the chemical stability and also enhancing the SLP value by tuning its magnetic properties. One such representation is a core@shell type nanostructure; where the presence of shell assists in tuning the magnetic properties of the core material due to their interface exchange coupling. Also; it is significant to account that, the presence of the shell not only tunes the magnetic properties but it amplifies the stability of the core material as well^13^. Another distinctive property originating from interface coupling is the exchange bias effect which manifests in enhancing coercivity and repositioning of the hysteresis loop towards the field direction^14–17^. Magnetic nanostructure exhibiting EBE has a great advantage in the field of biomedical applications as they can well stabilize and control in opposition to thermal fluctuations^18^. Presently, researchers are putting diverse attention to developing the interconnection between the EBE and the heating efficiency of MNPs. Recently, it was experimentally reported that the SLP value emerges proportionally to the strength of EBE value i.e. with a reduction in the strength of EBE; the SAR is hindered to a lesser value and vice-versa. Also, they have reported that an inverted CS counterpart is superior to the normal CS nanostructure endorsing to a better heating capability^19^. The magnetic interchange coupling at the interface of the CS nanostructure magnifies the heating ability of MNPs, no doubt. However, it is important to know as to which factor dominates more in the collaboration tuning of the magnetic properties through the coupling of core and shell material. Many theoretical, as well as experimental works have been executed and it is evident that the variation of the shell thickness is more significant in tuning the magnetic properties rather than performing a variation on the core thickness^20,21^. Essentially, the heating aptitude of the MNP relies upon sundry ingredients such as effective anisotropy, particle size, and distribution, saturation magnetization, applied field and frequency, the viscosity of the solvent, etc^22–25^. As described above, the shell performs a significant role in tuning the properties of the MNPs, and therefore; in this present work, we have chosen Fe_3_O_4_ as the shell material and Co_0.5_Zn_0.5_Fe_2_O_4_ as the core material, where the CS (Co_0.5_Zn_0.5_Fe_2_O_4_@ Fe_3_O_4_) nanostructure is carefully produced by making use of co-precipitation method. Eventually, an investigation to bare CoFe_2_O_4_ and CoFe_2_O_4_@Fe_3_O_4_ (or Fe_3_O_4_@CoFe_2_O_4_) is widely explored, while to our comprehension, Co_0.5_Zn_0.5_Fe_2_O_4_@Fe_3_O_4_ CS nanostructure has not been scrutinized for magnetic hyperthermia applications. In this toil, we have performed a comparative study of both bare F and CZ material with the CS CZF material and it resulted to better heating efficiency for CS in comparison to single materials that remarkably entitled the sample as a befitting agent for magnetic hyperthermia applications.

## Experimental

Ferric chloride (FeCl_3_.6H_2_O) (97%), Zinc chloride (ZnCl_2_) (99.5%), ferrous chloride (FeCl_2_.4H_2_O) (98%), Sodium hydroxide (NaOH) (80%) and Cobalt chloride (CoCl_2_·6H_2_O) (97%) were utilized to prepare the CS sample Co_0.5_Zn_0.5_Fe_2_O_4_@Fe_3_O_4_. All the raw materials were acquired from merk and employed without further moderation.

A two-step co-precipitation strategy was employed to synthesize the CS Co_0.5_Zn_0.5_Fe_2_O_4_@Fe_3_O_4_ MNPs. At first, an amount of 1.57 g of FeCl_3_·6H_2_O, 0.806 g of CoCl_2_·6H_2_O, and 0.849 g of ZnCl_2_ were liquefied at 50 mL Milli Q water and applied constant stirring for 30 min at 50 °C to homogenize the mixture. Also, during constant magnetic stirring, a solution of 1.07 g of NaOH dissolved in 15 mL Milli Q water was added drop-wise as a precipitating agent until pH was acquired to 12. After this, the magnetic solution was then heated and stirred continuously for 60 min for a suitable reaction at 80 °C. Then, a dark brown precipitate was cleansed multiple times with Milli Q water and ethanol for residual chloride removal. Lastly, the precipitate was kept at 80 °C for 12 h to dry where CZ MNPs were formed. In the following step, 3.12 g of FeCl_3_·6H_2_O, 1.24 g of FeCl_2_·4H_2_O, and 1 g of the already formed CZ grain powder were mixed in 50 mL Milli Q water to secure a certain definite molar concentration, followed by the same procedure described earlier in the first step. Finally, after washing several times with Milli Q water and ethanol, the sample was dried in the oven at 80 °C for 12 h and the dark brown sample was ground to fine powders by making use of a pestle and agate mortar, where CS CZF sample was acquired.

## Result and discussions

### XRD analysis

**Figure 1:**
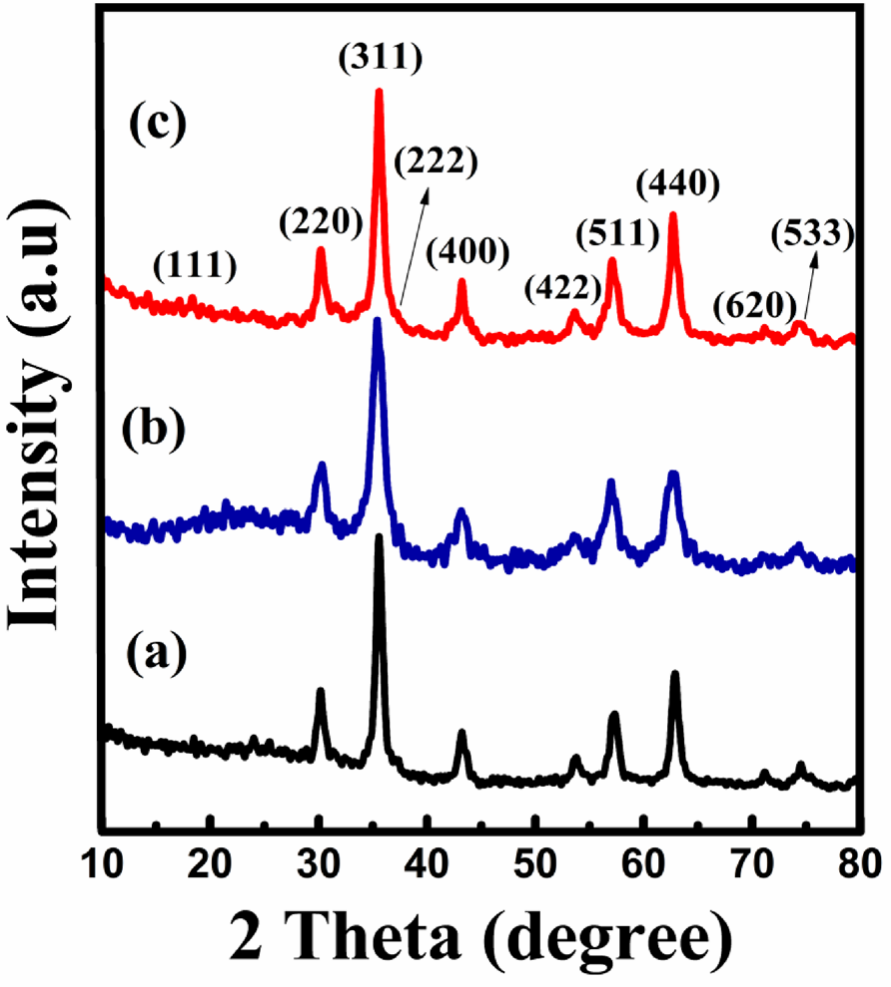
XRD spectra of (**a**) F, (**b**) CZ, and (**c**) CZF samples.

The XRD spectra manifested in Fig. 1a–c explicate the crystal structure and the vindication of crystal phase purity for (a) bare F, (b) bare CZ, and (c) CS CZF samples, respectively. The XRD peaks emanated for all three samples ensure an fcc structure which is in concurrence with the standard JCPDS cards; 22-1086 with Fd3mspace group for C sample, 89-3854 and 89-1010 for F and Z, both endorsing an Fd$\overline{3}$m space group, respectively. No other supplementary peaks are observed in the XRD spectra expounding the crystal phase purity of the as-synthesis samples. The FWHM(Full-Width Half-Maxima) of the fiercest peaks of the spectra were optimized, through which the mean crystallite sizes were estimated using Scherrer’s formula^26^. The evaluated mean crystallite size and the respective lattice constants for all the samples are lay-out in Table 1.

**Table 1:** Crystallite size and lattice constant of F, CZ, and CZF samples.

Sample	Crystallite size (nm)	Lattice constant (Å)
F	4.57	8.39
CZ	3.21	8.37
CZF	5.75	8.35

### Microstructure analysis

**Figure 2:**
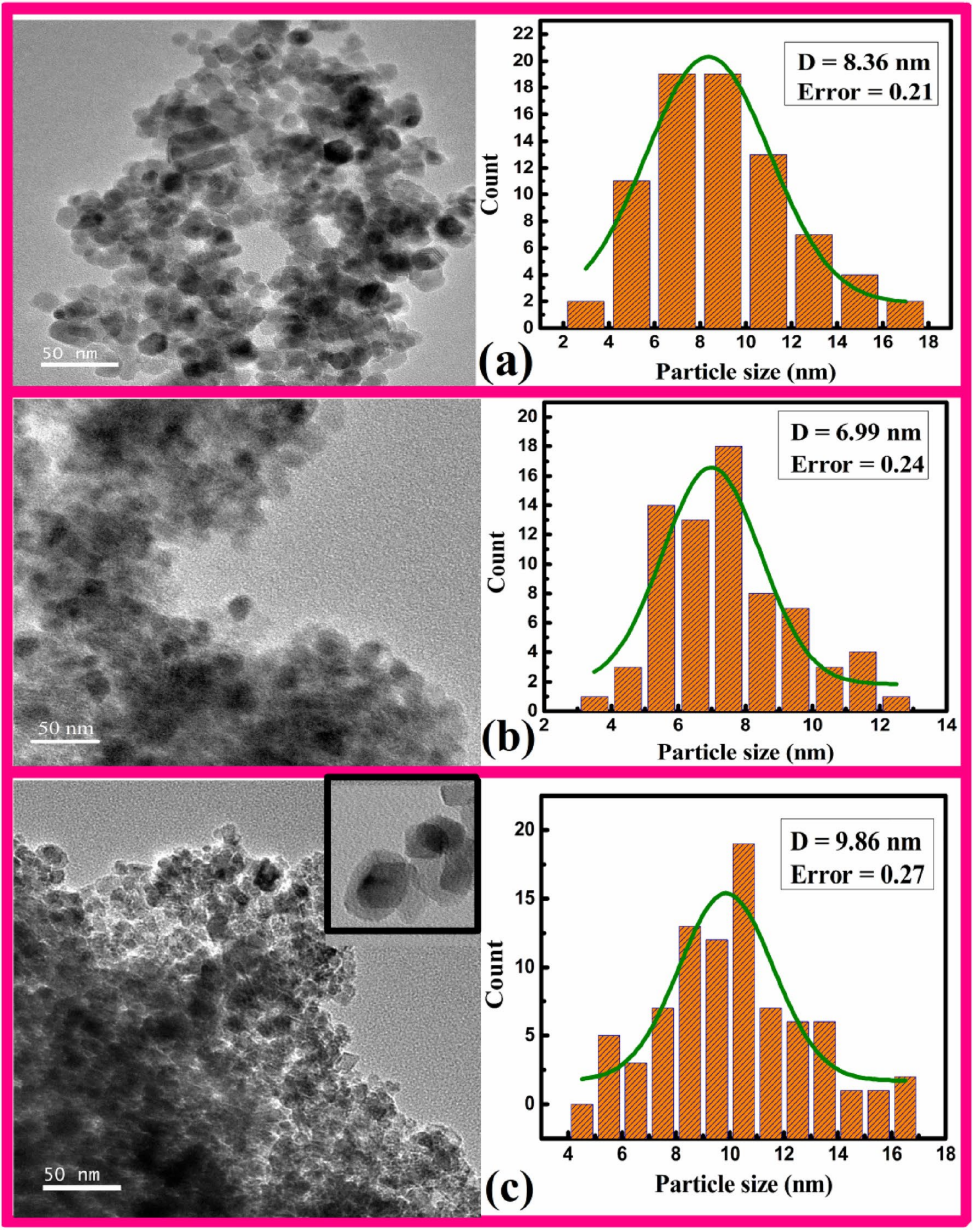
TEM micrograph with average particle size plotted histogram of (a) F, (b) CZ, and (c) CZF MNPs.

Figure 2a–c lay out the typical TEM micrographs along with their respective plotted particle sizes histogram for samples F, CZ, and CFZ, respectively. Figure 2a,b ensues a non-uniform distribution of MNPs with spheroids shape for the bare samples F and CZ, while the CS sample displayed in Fig. 2c exhibits similar shape but agglomerated NPs. Agglomeration of NPs might occur through the Brownian mechanism where the NPs are attached under their random motion and also due to the gravitational effect which is dependent on the thermal speed and the particle size^27^. However, the TEM image along with the inset shown in Fig. 2c authenticates a CS nanostructure. The average associated particle sizes obtained through the fitted histogram are 8.36 ± 3 nm, 6.99 ± 2 nm, and 9.86 ± 3 nm for F, CZ, and CZF respectively. The enhanced particle size for the CS sample might result due to higher polydispersity originating through the second step synthesis procedure involved^28^. The average shell thickness for the CS sample is evaluated with its mean size of about 1.89 ± 0.32 nm.

**Figure 3:**
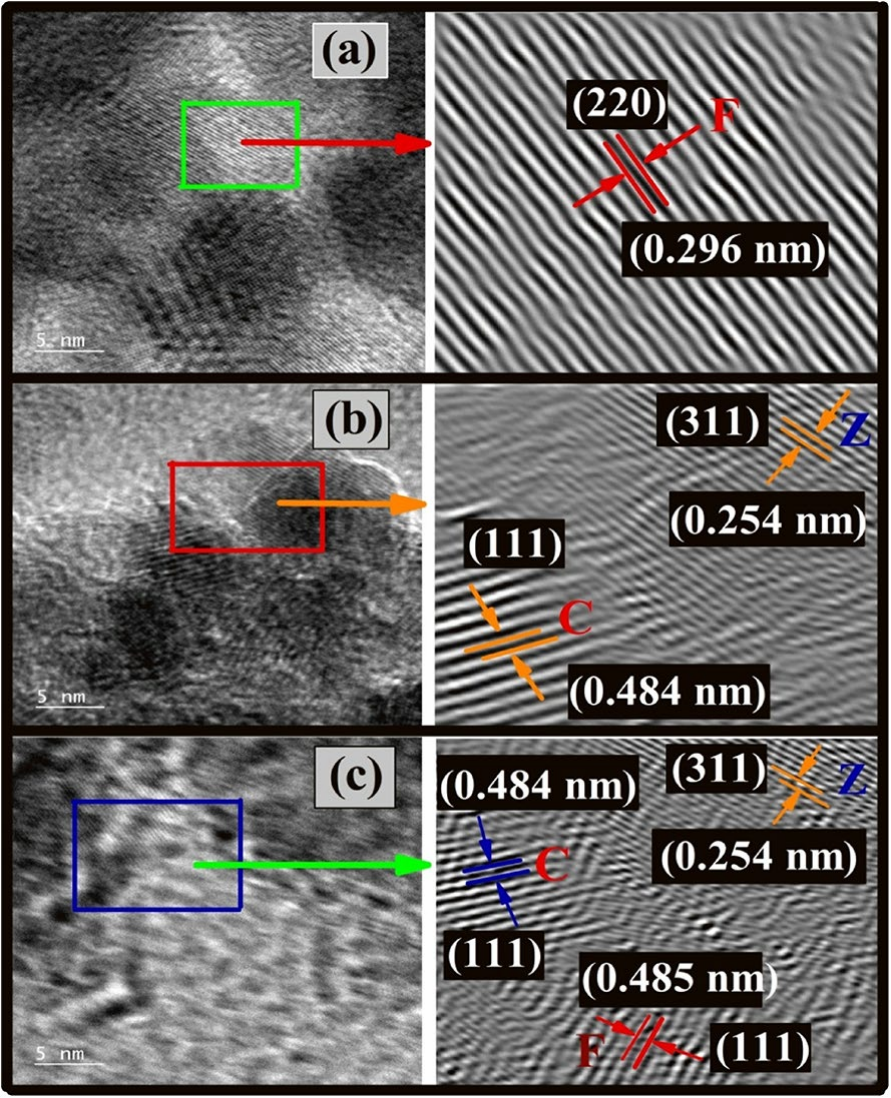
HRTEM image with IFFT lattice planes micrographs of (**a**) F, (**b**) C, and (**c**) CZF MNPs.

Figure 3a–c present the HRTEM micrographs with their respective IFFT (Inverse Fast Fourier Transform) images validating the d-spacing value for F, CZ, and CZF samples. Figure 3a reveals the phase inclination for the (220) plane with its respective d-spacing value (d_220_ = 0.296 nm) for the bare F sample. Also, for the mixed ferrite CZ sample shown in Fig. 3b, the d-spacing value for C and Z are individually verified with the (111) plane for C (d_111_ = 0.484 nm) and (311) plane for Z (d_311_ = 0.254 nm), respectively. Further, for the CS sample CZF, the IFFT image was extracted from the premise of the CS interface capturing all the planes for C, Z, and F. The crystal planes for core sample CZ is identical as described above in Fig. 3b, while the shell sample F is authenticated to d-spacing value (0.485 nm) for (111) crystal plane. All the evident d-spacing values for C, Z, and F are verified with their respective JCPDS file no. describe in the XRD analysis.

**Figure 4:**
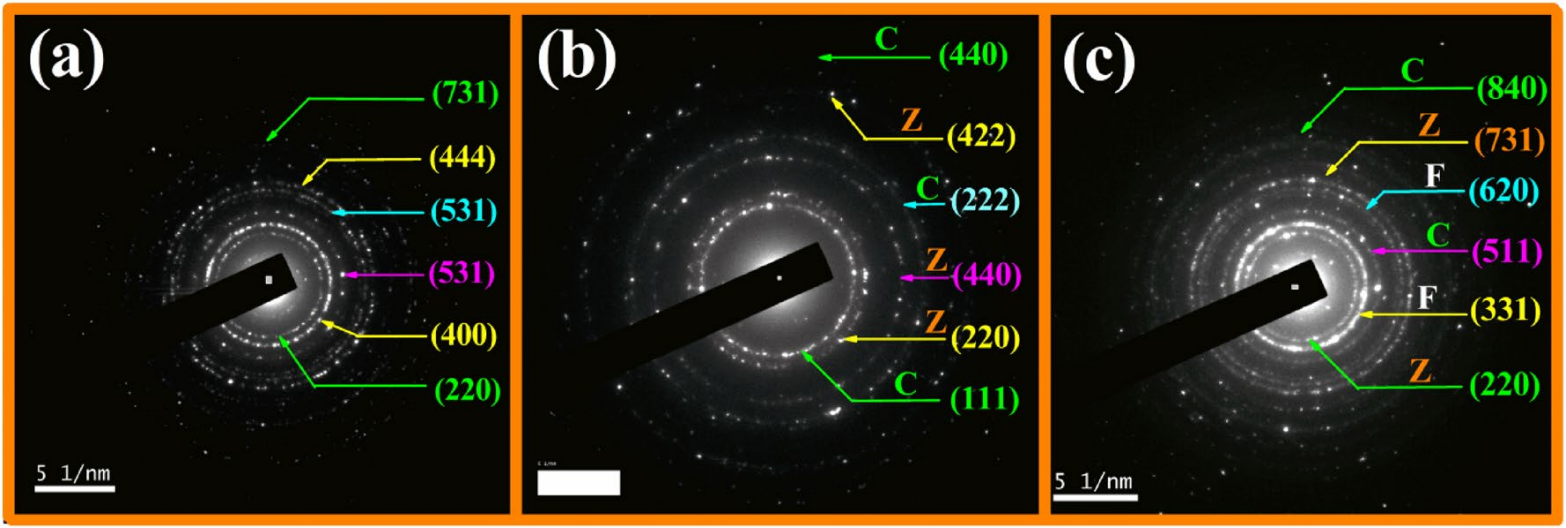
SAED pattern of (**a**) F, (**b**) CZ, and (**c**) CZF MNP.

Figure 4a–c convey the SAED spectra achieved by the MNPs displaying concentric rings divulging to its MNPs polycrystalline nature. The SAED diffraction rings secured for bare shell (F) and core (CZ) samples disposed of in Fig. 4a,b authenticate to their respective crystal plane orientation complementing the XRD analysis. Further, the SAED spectra survey for the CS sample CZF expressed in Fig. 4c distinctly disclosed the coexistence of both the crystal planes of core and shell samples. The electron diffraction rings perceived owing to planes (331) and (620) communicate to shell material F. However, the concentric rings on account to crystal planes (511) and (840) correspond to C material, while the rings due to crystallographic planes (220) and (731) are owing to Z material of the core sample CZ, which reflects authentically to XRD results.

**Figure 5:**
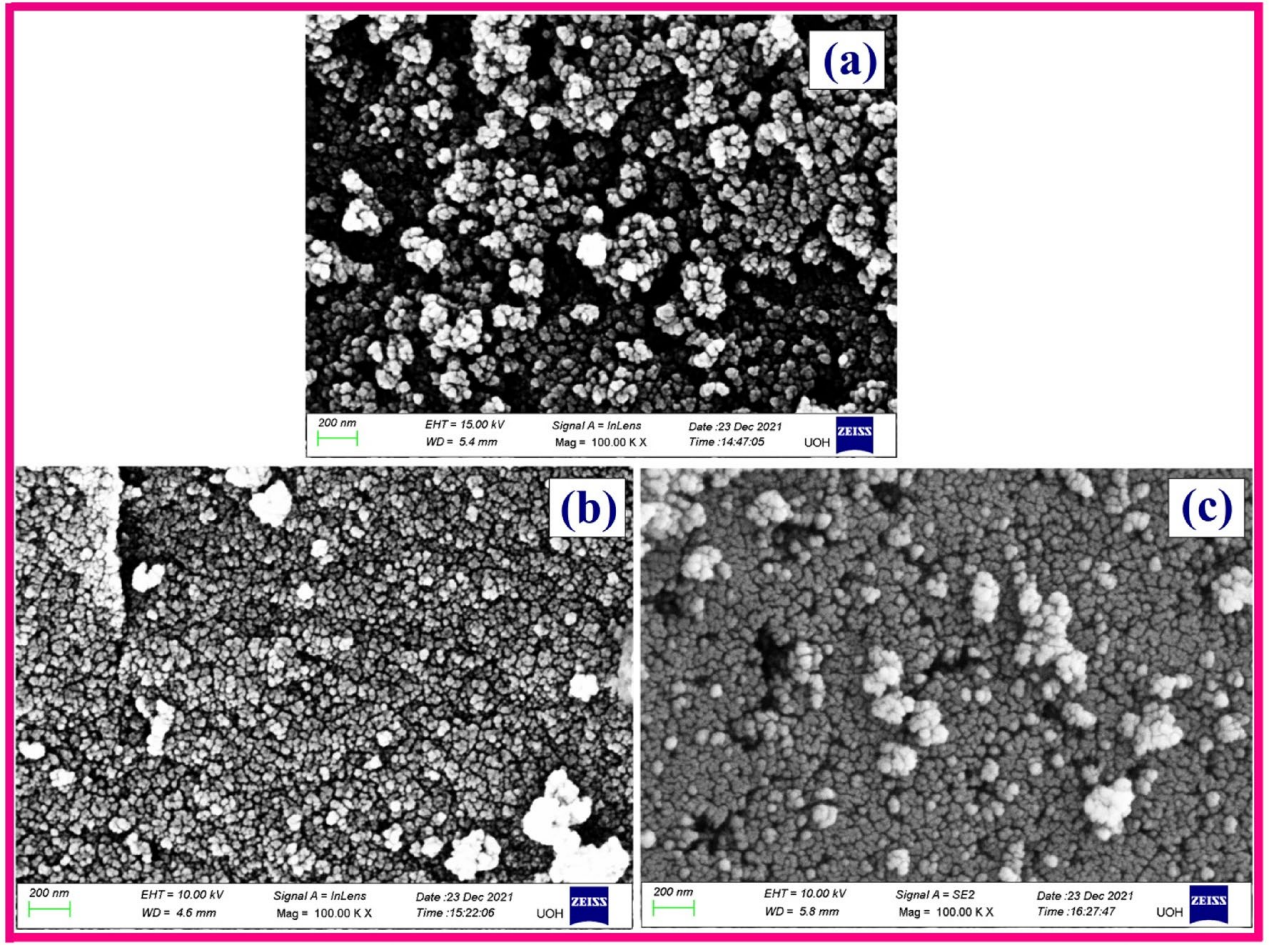
FESEM micrographs of (**a**) F, (**b**) CZ, and (**c**) CZF MNP.

An extensive inspection to morphology of the samples were further carried out by engaging FESEM, where the visualized morphology images of F, CZ, and CZF samples are shown in Fig. 5a–c, respectively. As displayed in the figures, an agglomeration is slightly revealed for all the samples exhibiting a spheroids shape particles similar to the images obtained from TEM analysis. The presently observed morphologies of F and CZ samples are identical to the morphology of the previously reported literature for the same synthesis method employed^29,30^. The average particle diameter observed through the FESEM analysis for F, CZ, and CZF samples are respectively 15.32 ± 2 nm, 12.76 ± 3 nm, and 18.21 ± 2 nm. The sequence of particle sizes obtained from FESEM examinations is in concurrence with the TEM result. The enlargement particle size secured for the CS CZF sample might regard to the fine growth of F nanoparticles as a shell on the surface of the core CZ material.

**Figure 6:**
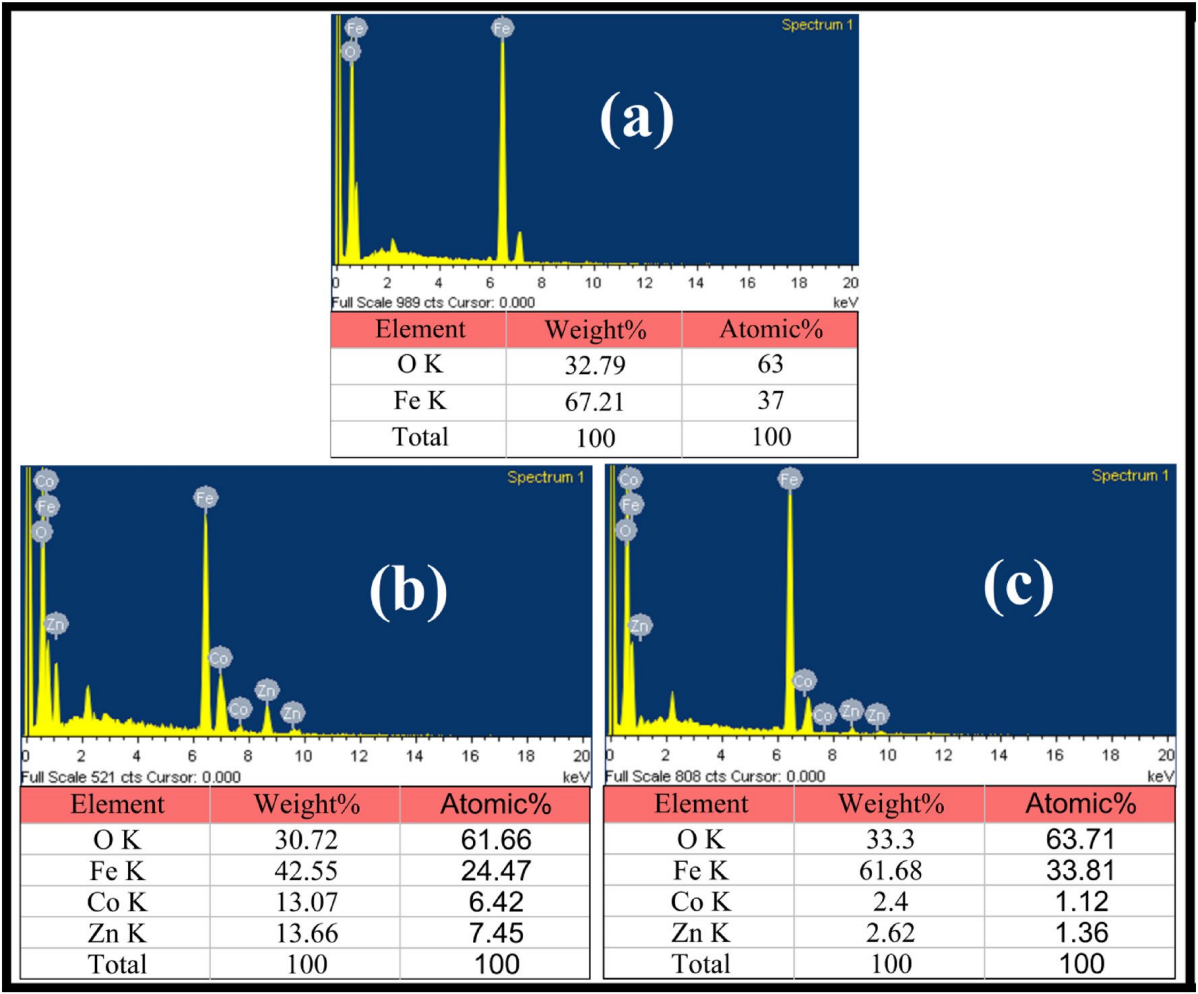
EDX patterns of (**a**) F, (**b**) CZ, and (**c**) CZF samples.

The elemental composition of all the respective samples was detected utilizing an energy dispersive X-ray (EDX) analysis, where the corresponding spectra along with their elemental composition ratio in terms of atomic and weight percentage (%) are depicted in Fig. 6a–c. The peaks observed in Fig. 6a are on account of elements Fe and O which authenticate the origination of pure F crystal. Also, the EDX peaks perceived in Fig. 6b are divulging for elements Fe, O, Co and Zn respectively, revealing the emergence of a pure CZ material. The elemental component acquired in Fig. 6c for the CZF sample is identical to that of the CZ sample. However, the elemental proportion ratio in weight% and atomic% are unlike as visualized in the figure. The enhanced component of Fe elements achieved for the CS CZF sample might regard to the presence of F material as a shell component in the CS sample. The elemental composition ratio in % observed for all the samples are commensurate to the experimental anticipated % which is evident that the disappearance of elements is not discerning during synthesis.

### FTIR analysis

**Figure 7:**
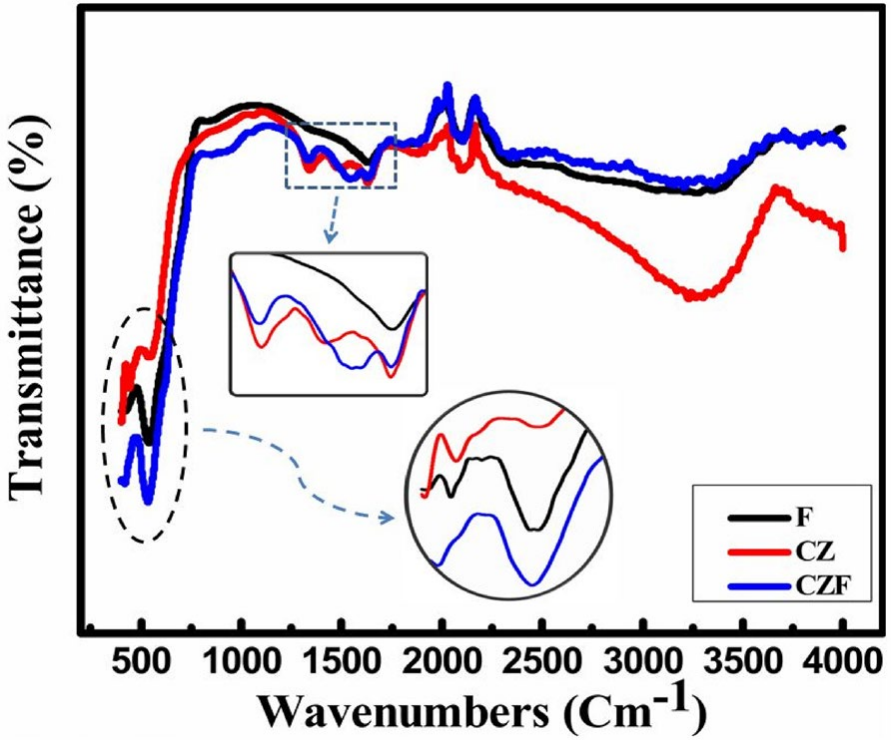
FTIR transmission spectra for F, CZ, and CZF samples.

Figure 7 unveiled the FTIR spectroscopy analysis recorded at the 400–4000 cm^−1^ range to recognize the distinct vibrational characteristics of the lattice absorption bands for F, CZ, and CZF, respectively. As displayed in Fig. 7, all the distinct characteristic peaks for bare samples F and CZ are present at the CS sample CZF except that a slight shifting is recorded for the same. The probable reason leading to the shifting of peak position for CS CZF might accredit to the substitution of cobalt ions with Fe ions appearing in the octahedral sites, noting that the ions have unlike atomic mass and ionic radii^31^. Usually, the absorption bands exhibiting below 1000 cm^−1^ correspond to spinel ferrite features attributing to metal–oxygen (M–O) stretching vibrations^32,33^. The absorption peak spotted at 539.28 cm^−1^ for the F sample is allocated to the stretching vibrations of Fe^2+^ and O^2−^ ions. Also, the two strong IR bands perceive around 400–500 cm^−1^ and 500–600 cm^−1^ for both CZ and CS CZF samples corresponding to intrinsic M–O stretching vibrations in the tetrahedral and octahedral sites^34–36^. Therefore, the recorded IR peaks corresponding to M–O vibration modes divulge the origination of spinel ferrite cubic NPs.

### Magnetic study

**Figure 8:**
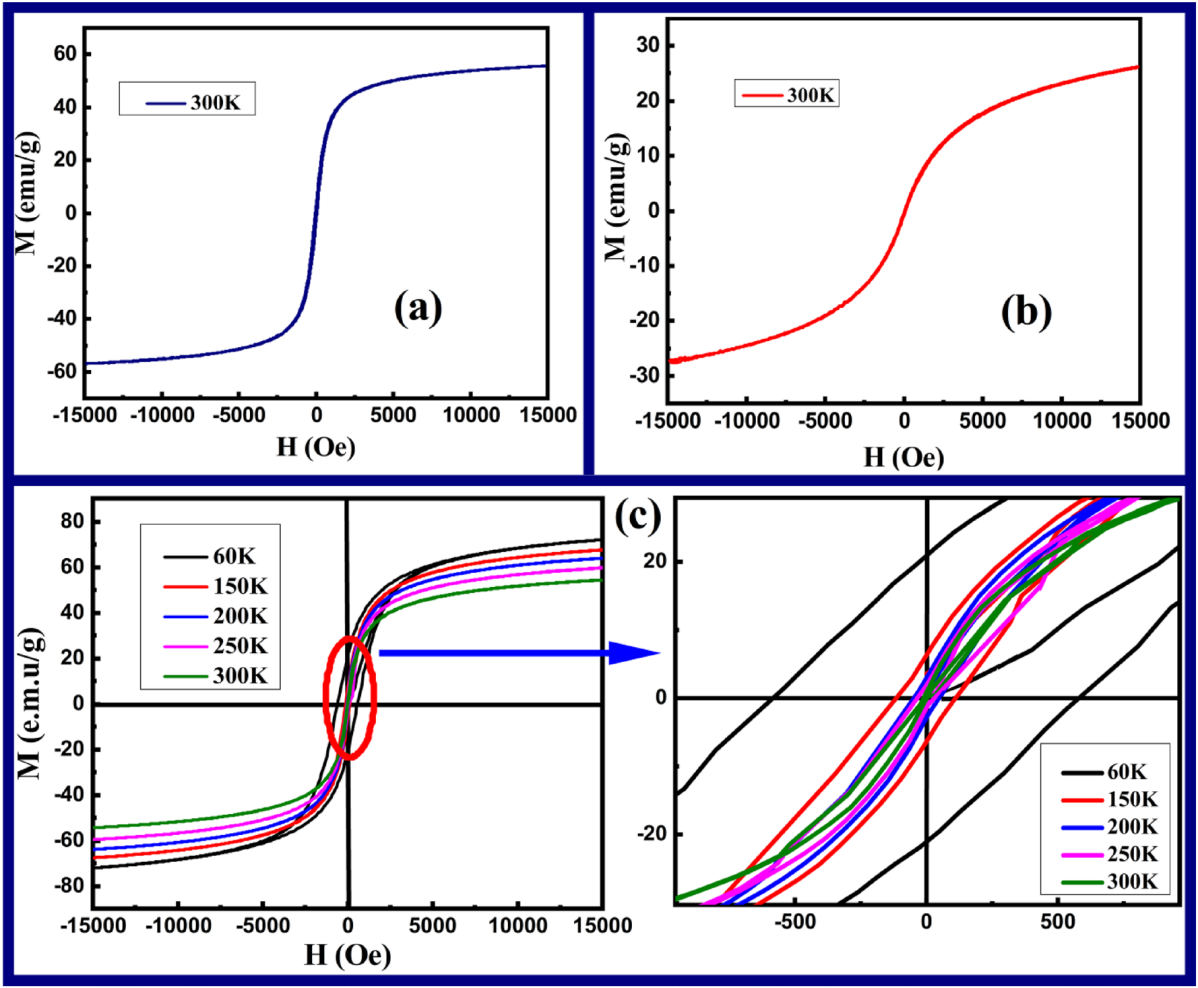
Magnetic hysteresis loop (**a**) at 300 K for F, (**b**) at 300 K for CZ samples, and (**c**) five distinct temperatures for CS CZF sample.

**Figure 9:**
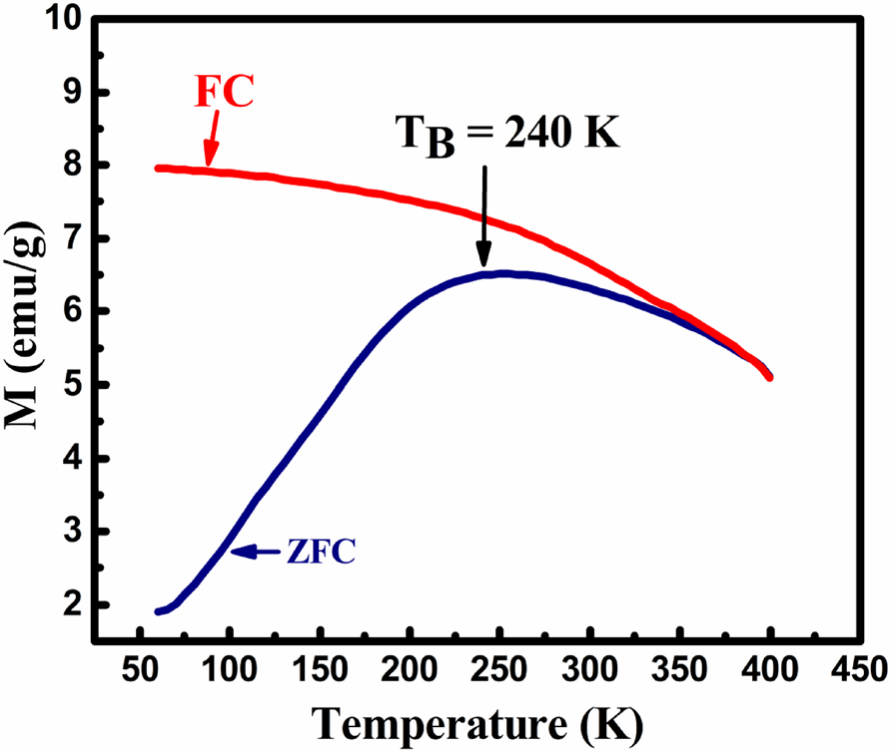
ZFC–FC plot at 100 Oe for CS CZF sample.

The magnetic quantification of MNP over applied field and temperature were examined through Physical Properties Measurement Systems (PPMS) and the emanated outcomes are depicted in Fig. 8a–c. The M-H loops perceived at room temperature (300 K) for single materials F and CZ are manifested in Fig. 8a,b, while the M-H hysteresis loops acquired for CS CZF sample at five distinct temperatures (60 K, 150 K, 200 K, 250 K and 300 K) are shown in Fig. 8c. The saturation magnetization (*M*_*S*_) for samples F, CZ, and CZF at room temperature is achieved at about 57.34 emu/g, 27.89 emu/g, and 52.65 emu/g, respectively. The *M*_*S*_ value for the CS CZF sample is a joint benefaction made by both CZ (core) and F (shell) samples. However, the value leans more towards the F value which might be attributed to the more magnetic contribution made by the shell material. Interestingly, other magnetic parameters such as coercivity (*H*_*C*_) and retentivity (*M*_*R*_) follow the same fashion for the CS CZF sample over bare F and CZ samples. Thus, the outcome proclaims that the core and shell materials are coupled magnetically at their junction interface^28,37,38^. The magnetic anisotropy (*K*_*C*_) of F, CZ, and CZF samples were appraised by employing the law of approach fitting through initial magnetization curves^39^. All the magnetic parameters perceived at room temperature are specified in Table 2. A low-temperature survey on the M-H loop was further employed for the CS CZF sample, for which an enhancement to saturation magnetization and coercivity is apparent at lowering the temperature. This implies that more magnetic moments tend to orient along the applied field attributing to more dominance of the magnetic field over temperature^38,40^. To the utmost interest, the inflation in the *M*_*S*_ and *H*_*C*_ at lowering temperature is also escorted by loop relocation along the negative axis ascribing to the phenomenon of EBE. Generally, the relocation of the hysteresis loop originates as a consequence of induced unidirectional anisotropy engender through the interface exchange coupling between the core and shell materials^41,42^. Although, the macroscopic origin of EBE exists for more than 50 years and despite its vital implementation in multiple applications, the microscopic behavior or its coupling mechanism is yet to fully recognize, which is still a debated field of interest. However, it is definitive that the EBE arises solely due to a small fraction of spins adheres at the interface layer of the core and shell materials despite its different combinations of core and shell systems (i.e. a system composed of Ferro/ferri-magnetic@anti-ferromagnetic,anti-ferromagnetic@ferro/ferri-magnetic, Ferro/ferri-magnetic@ferro/ferri-magnetic, etc.).It was experimentally evinced by Ohldag et al. that the induced unidirectional anisotropy generated through interface coupling is out-turn significant by a very small fraction less than about 4% of aligned interfacial spins at the junction of core and shell layer^43^. Therefore, the consequence of such frustrated spins might have been initiated through the frozen spins at the interface which might be the factor responsible for the interface exchange anisotropy (EBE), although it provides very few donations towards the total magnetization. Also, one should take note that, under field cooling, the uncompensated spins at the interface surface and shell layer which are pinned to alignment are other factors guiding to EBE phenomenon. When the interfacial spins are pinned, it will require a large amount of Zeeman energy to orient along its direction, and therefore, in an attempt to neutralize the system, a negative intrinsic field is generated leading to the relocation of the M-H loop along the negative axis. For the present samples, the minimum temperature employed to investigate the M-H loop is 60 K. However, EBE is authenticating even at 60 K perceiving its maximum value as compared to the observation made at higher temperatures. The exchange bias field (H_ex_) induced at 60 K is H_ex_ = − 10.34 Oe and the value shrinks as we further surge the temperature. The minimum H_ex_ is noticed at 200 K (H_ex_ = − 3.94 Oe) and no shifting is absolute at 250 K and 300 K. It is well understood that the shifting of the M–H loop usually fades away at the blocking temperature^44,45^. Therefore, for further authentication, we have performed ZFC–FC measurement for the CS CZF sample at the cooling field of 100 Oe, where the plotting is displayed in Fig. 9. It is obvious from the figure that, the CZF sample perceived a blocking temperature of about 240 K which provides a complete picture of the non-existence of EBE at 250 K. Therefore, from the outcome resulted, it may be understood that more interfacial spins are pinned to alignment at lower temperature directing to higher H_ex_. However, as the temperature gets enhanced, the thermal energy suppresses the local anisotropy and allows the interfacial spins to an easier orientation towards the field direction, thus producing hindrance to EBE, which finally disappears at the blocking temperature and beyond which no relocation is evident. The magnetic properties of CS CZF at lower temperatures are designated in Table 3.

**Table 2:** Magnetic parameters for F, CZ, and CZF samples at 300 K.

Sample	M_S_ (emu/g)	M_R_ (emu/g)	H_C_ (Oe)	M_R_/M_S_	K_C_ (× 10^4^ Jm^−3^)
F	57.34	3.21	50.14	0.055	4.87
CZ	27.89	0.18	21.48	0.006	1.69
CZF	52.65	1.31	37.25	0.032	3.42

**Table 3:** Magnetic properties of CS CZF sample for different temperatures.

Temperature	M_S_ (emu/g)	M_R_ (emu/g)	H_C_ (Oe)	M_R_/M_S_	H_ex_ (Oe)
250 K	63.11	2.12	42.55	0.034	–
200 K	67.70	2.61	49.23	0.039	− 3.94
150 K	71.96	6.4	113.21	0.089	− 5.83
60 K	76.23	21.16	582.49	0.278	− 10.34

### Induction study

**Figure 10:**
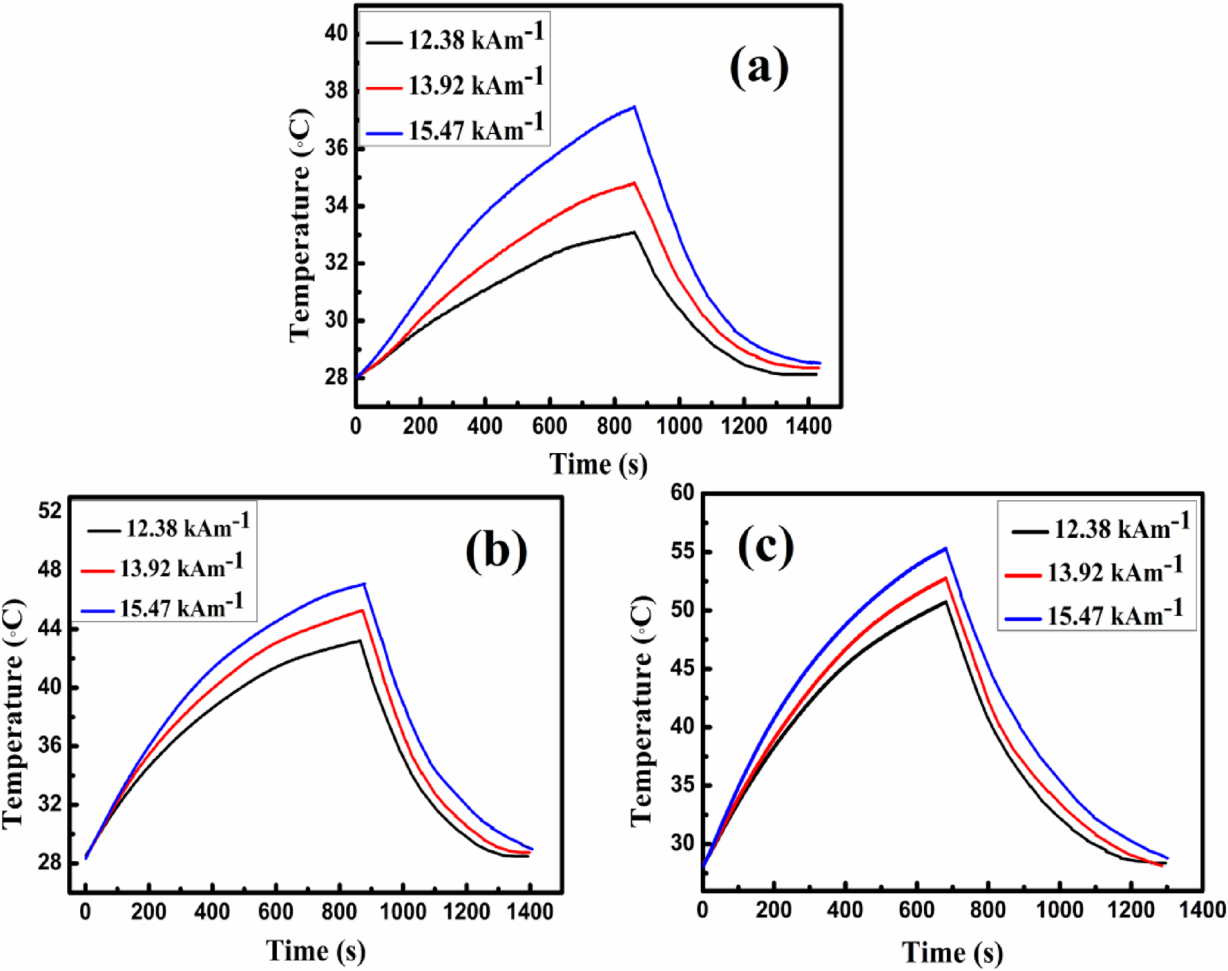
Heat dissipation with time and cooling curve for (**a**) CZ, (**b**) F, and (**c**) CZF MNPs at three different fields.

The rate of temperature inflation with time for the MNP was analyzed employing an easy heat 8310 (Ambrell make UK) having 7 and 8 turns of diameter coils dispatched at three distinct fields i.e. 12.38 kAm^−1^, 13.92 kAm^−1^ and 15.47 kAm^−1^ and conventional frequency (f = 337 MHz) for samples CZ, F, and CZF, respectively. This particular investigation is carried out to recognize the heat generation potential of the MNP which is quantified to a specific loss power (SLP). The prime objective is to acquire supreme SAR value with the least sample concentration within the clinical safety restraint called Brezovich limit^9^ i.e. the multiplication of frequency and the applied field should not surpass ≈ 5 × 10^9^ Am^−1^ s^−1^. In our study, the multiplication of frequency and the distinct applied fields (H × f) falls in less than the clinical restraint; thus satisfying one of the foremost requirement for hyperthermia applications. To optimize the heating perspective of the MNP, 1 mg mass of each sample (CZ, F, and CZF) were solvated in 1 mL Milli Q water which is further placed within the work-coil. Then the sample is placed in AC magnetic field with the particular amplitude and frequency, where the fibre optics temperature probe simultaneously records the temperature rise of the sample with respect to time for about 15 min (900 s) for all the three distinctive applied fields, where their respective results yielded are illustrated in Fig. 10a–c. An air cooled insulation and polyurethane coil was used between the nanoparticles tube and induction coil in order to secure the nanoparticles in being heated up by residual heat through the induction coil. The heating potential or the SLP value prevailed through a non-adiabatic setup, where the curve is contoured manoeuvring the Box–Lucas (B–L) equation^46,47^. It is noticed that the CZ sample does not acquire the hyperthermia threshold temperature (42–46 °C) even for the measurement recorded at 15.47 kAm^−1^ field. The impotence to acquiring threshold temperature for the CZ sample might regard to its low anisotropy energy and low saturation magnetization^48^. Sample F being associated with the highest saturation magnetization and anisotropy energy achieved the temperature requirement within 900 s for all the respective fields. Interestingly, the CS CZF sample executed to threshold temperature within 500 s despite its lower magnetic response compared to F materials. This outcome manifests that more heat dissipation prevailed for CZF MNP. The probable reason behind this output could be related to the EBE phenomenon resulting from the exchange coupling at the interface junction of the core and shell materials, where the benefaction of this additional interface exchange anisotropy energy eccentrically tunes the effective anisotropy of CZF sample^49–51^. The wielding of suitable shell material with appropriate thickness is another prime component in magnifying the surface anisotropy which is an additional endowment to the total effective anisotropy. Moon et al*.* experimentally delineate that, Fe_3_O_4_ with a thin shell to about ≈ 1–2 nm plays a crucial part to enhance the surface anisotropy that contributes to the higher heating capability of the MNP^52^. The SLP values secured at 12.38 kAm^−1^ field are 43.79 W/g, 160.59 W/g, and 276.08 W/g for the corresponding samples CZ, F, and CZF, respectively. The inflation on the applied magnetic field will pilot the magnetic spins to more fluctuations that will engender lofty heat dissipation of the MNP. Consequently, the highest SLP value is secured for the measurement drawn at 15.47 kAm^−1^ field for the CS CZF sample which is acquired to about 340.85 W/g. It is also supreme to obtain a normalized value for the corresponding SLP values through the removal of the extrinsic parameters since the power inception of the MNP scales linearly with the frequency and quadratically to the magnetic field applied, which is regarded as intrinsic loss power (ILP)^53,54^. The calculated ILP values and the corresponding SLP values for all the respective currents are prescribed in Table 4. It is also cardinal to note that, the typical ILP values reported in the earlier literature^55–58^ for suitable heating take a value ranging between 3 and 4 nHm^2^ g^−1^. Our perceived ILP values for the CS CZF sample are beyond 4 nHm^2^ g^−1^, with the highest recorded for 12.38 kAm^−1^ field which is about 5.36 nHm^2^ g^−1^ remarking as a suitable agent with high heating capability. Also, as shown in Fig. 10, on removal of the applied magnetic field, the nanoparticles cooled down to its initial temperature taking just about 10 min approximately.

**Table 4:** Values of ILP and SAR at three different fields for all the samples.

Samples	SLP (W/g) for 1 mg			ILP × 10^−3^ (nHm^2^ g^−1^)		
	12.38 kAm^−1^	13.92 kAm^−1^	15.47 kAm^−1^	12.38 kAm^−1^	13.92 kAm^−1^	15.47 kAm^−1^
CZ	43.79	56.02	81.88	0.85	0.86	1.01
F	160.59	185.99	218.48	3.12	2.96	2.61
CZF	276.08	289.71	340.85	5.36	4.45	4.24

Therefore; based on the MNP heating retaliations, it may be pointed out that for the CS CZF sample, the employment of befitting shell material with suitable thickness amplifies the surface anisotropy and the manifestation of extra interface exchange anisotropy as a consequence of the EBE through the junction interface coupling had significantly modulated the effective anisotropy, thus directing to the superior heating capability of the CS sample. Hence, it is commended that a core@shell type nanostructure is genuinely prominent to effectively revamp the heating capability of the MNP, and therefore, the CS CZF sample is marked to be a desirable emissary to effectual magnetic hyperthermia applications.

## Conclusion

CZ, F, and CS CZF MNP samples were meticulously prepared by utilizing a co-precipitation course of action, where the investigation on its microstructure, morphology, and magnetic behavior was carried out employing an XRD, FESEM, HRTEM, and VSM, respectively. The origination of CS nanostructure is apparent through the TEM micrograph, where a slightly agglomerated spheroids MNP is visualized through the FESEM and TEM micrographs. A lattice plane authentication through the HRTEM study further confirmed the core and shell materials, where their elemental compositions are divulged employing an EDX. The VSM appraisal disclosed a relocation of the hysteresis loop (EBE) for the CS CZF sample, substantiating to strong exchange interaction between the core and shell materials. It is also validated that, the EBE fades away at the blocking temperature (T_B_ = 240 K). Intriguingly, magnetic response to saturation magnetization and anisotropy is higher for the F sample; however, the heat liberated by the CS CZF MNP was outrageously amplified compared to both CZ and F samples reaching hyperthermia threshold temperature within 500 s. The credible reason behind this astonishing consequence might regard to the usage of appropriate soft shell material with a befitting thickness that remarkably ameliorates the surface anisotropy and also, the significant outcome of EBE legitimating to hefty exchange coupling at the interface of the core and shell materials bestowing to extra interface exchange anisotropy, which consequentially tuned the effective anisotropy of the CS CZF sample. A notably high SLP value of about 340.85 W/g with a lofty ILP value is attained for the CS sample. Therefore, CS nanostructure with astonishing interface coupling phenomenon (EBE) is conspicuous to intensify the heating ability of the MNP, and hence, the CS CZF sample is a prominent agent to contrivance on magnetic hyperthermia applications.

## Data Availability

The datasets generated and/or analyzed during the current study are available in the crystallographic open database repository, [COD ID: 1010369 and 1010130].
